# Hydatid Disease of the Lung

**Published:** 1904-07-30

**Authors:** 


### HYDATID DISEASE OF THE LUNG.

A case of this unusual disease is reported by Miss Aldrich Blake, M.D.[1] The patient, a woman of 51 years, had complained for some months of cough, with expectoration and loss of flesh. There was dull percussion and deficient respiratory murmur over the greater part of the right back, but exploration failed to detect any pleural effusion. A skiagram revealed a dark shadow in the right lung with a convex upper border at the level of the third rib. Hydatid hooklets were found in the sputa. Under chloroform portions of the seventh and eighth ribs were removed and the cyst evacuated. The contents, consisting of 3 pints 18 oz. of daughter-cysts with very little fluid, were removed by finger forceps and gentle syringing. Some of the cysts in the lower part of the cavity were bile-stained, but no perforation of the diaphragm could be recognised. The subsequent progress of the case was uneventful. It is noted that nothing like a mother cyst was seen either at the operation or in the after-treatment of the case, and in explanation of this it is suggested that the mother cyst may have been quite small and that the main mass of the hydatid was an exogenous formation. The circumstances, of course, suggest that the original site of the disease was the liver, and that the thoracic condition was a secondary development. There is no doubt that such an order of events may occur without any positive evidence of hepatic disease, and the primary cyst may be small and beyond the reach of physical examination. The present writer has recently seen a case in which, on the view that the patient was suffering from gall-stone, the abdomen was opened, but with entirely negative results. Later, though the patient ceased to complain of pain, he developed a febrile temperature with sweatings and loss of flesh. For some time no explanation of these conditions could be discovered, but after several weeks signs of fluid effusion at the base of the right chest were detected. An exploratory needle obtained a purulent-looking fluid, and when a rib was resected the discharge contained numerous hydatid hooklets and daughter cysts. There can be little doubt that here the original condition was a hydatid cyst of the liver, which at first caused pain, perhaps by producing a limited diaphragmatic pleurisy or a localised perihepatitis. So inconspicuous was this cyst, presumably on account of its small size and its situation on the upper surface of the liver, that it escaped the observation of the surgeon at the exploratory operation. Subsequently the diaphragm was perforated and a suppurative pleurisy was established. The patient made an excellent recovery. Not only may hydatid disease of the liver exist without distinctive symptoms and the condition fail to be recognised until extension to the chest has occurred, but an equal condition of quiescence may obtain in hydatid disease of the lung. This is probably very exceptional, but it occurred in a case reported by Dr. F. J. Smith.[2] The patient was a boy in whom there were no physical signs and no symptoms worth mentioning, yet he is described as having coughed up the whole of a hydatid cyst in fragments. All these cases illustrate the necessity of bearing in mind the possibility of hydatid disease in cases of more or less obscure disease within the thorax.

[1] Brit. Med. Jour., July 23, 1904. [2] Lancet, February 9, 1901.

